# Estrogen-Related Receptor γ Induces Angiogenesis and Extracellular Matrix Degradation of Temporomandibular Joint Osteoarthritis in Rats

**DOI:** 10.3389/fphar.2019.01290

**Published:** 2019-11-06

**Authors:** Haoming Zhao, Shaopeng Liu, Chuan Ma, Shixing Ma, Guokun Chen, Lingyu Yuan, Lei Chen, Huaqiang Zhao

**Affiliations:** ^1^Shandong Provincial Key Laboratory of Oral Tissue Regeneration, School of Stomatology, Shandong University, Jinan, China; ^2^Department of Oral and Maxillofacial Surgery, School of Stomatology, Shandong University, Jinan, China; ^3^Department of General Dentistry, Ningbo Stomatology Hospital, Ningbo, China; ^4^Department of Orthodontics, School of Stomatology, Shandong University, Jinan, China

**Keywords:** osteoarthritis (OA), temporomandibular joint (TMJ), estrogen-related receptor γ (ERRγ), extracellular matrix degradation, angiogenesis, interleukin 6

## Abstract

The main causes of cartilage destruction during temporomandibular joint osteoarthritis (TMJOA) are extracellular matrix degradation and angiogenesis, accompanied by an increased level of matrix-degrading enzymes and proangiogenic factors. Interleukin 6 and extracellular signal–regulated kinase (ERK) signaling pathways may play a critical role in these two processes simultaneously, but researchers have not clearly determined the mechanism. We hypothesized that estrogen-related receptor γ (ERRγ) is involved in both cartilage degeneration and angiogenesis in TMJOA. The interactions between ERRγ and the *Mmp9* and *Vegfa* promoter regions were investigated using a chromatin immunoprecipitation (ChIP) assay. A chick embryo chorioallantoic membrane (CAM) assay was performed to investigate the inhibitory effects of U0126 and GSK5182 on angiogenesis. Western blotting, reverse transcription–quantitative PCR (RT-qPCR), immunofluorescence staining, toluidine blue staining, and transfection with cDNAs or small interfering RNAs (siRNAs) were performed on primary mandibular condylar chondrocytes (MCCs). Unilateral anterior crossbite–induced TMJOA models were established in rats, and Western blotting, RT-qPCR, immunohistochemistry, and Safranin O-Fast Green staining were performed to evaluate changes *in vivo*. ERK1/2 activated matrix metalloproteinase 9 (MMP9) and vascular endothelial growth factor A (VEGFA), which are involved in cartilage destruction, through ERRγ. Based on the ChIP assay results, ERRγ directly activated the transcription of the *Mmp9* and *Vegfa* genes. In chick embryo CAM models, U0126 and GSK5182 significantly inhibited angiogenesis. In conclusion, ERRγ is a downstream transcription factor of ERK1/2, and its upregulation leads to extracellular matrix degradation and angiogenesis in TMJOA. This study identified a common factor between inflammation and vascularization in OA as well as a new therapeutic target for OA: ERRγ.

## Introduction

Osteoarthritis (OA) is a whole-joint disease characterized by cartilage matrix destruction, subchondral bone sclerosis, osteophyte formation, synovitis, and articular cartilage absorption (Li et al., 2018). Cartilage vascularization and synovial angiogenesis are also important pathological features (Mapp and Walsh, 2012). Although articular cartilage is an avascular tissue (Brucker et al., 2005), when OA occurs, increased angiogenesis in the synovium, articular disc, and resulting callus lead to osteophyte formation and deep ossification of the joint (Brown and Weiss, 1988; Franses et al., 2010). Unfortunately, effective disease-modifying therapies for OA are not available. The mandibular condylar chondrocyte (MCC) is a specialized functional cell of the temporomandibular joint (TMJ) (Chen et al., 2014), and a number of TMJ diseases are caused by damage to these cells (Zhou et al., 2015). Matrix metalloproteinase 9 (MMP9) (Ahmad et al., 2018), and vascular endothelial growth factor A1 (VEGFA) (Shen et al., 2015) play crucial roles in cartilage destruction and synovial angiogenesis in subjects with temporomandibular joint osteoarthritis (TMJOA) because these proteins are expressed at high levels in MCCs.

Interleukin 6 (IL-6) is a member of a family of cytokines known as gp130 cytokines and has been shown to be responsible for various musculoskeletal disorders, particularly rheumatoid arthritis (RA) (Singh et al., 2016). IL-6 binds to its nonsignaling specific receptor (IL-6R) and common subunit (gp130) to activate two main signaling pathways: extracellular signal–regulated kinase (ERK) and signal transducer and activator of transcription (STAT) (Mihara et al., 2012). Matrix-degrading enzyme expression is upregulated through a process regulated by ERK phosphorylation, which is why the extracellular matrix is degraded in subjects with TMJOA (Ma et al., 2014; Zhao et al., 2015; Latourte et al., 2017; Khan et al., 2017). However, the specific mechanism by which ERK regulates MMP9 and VEGFA levels remains unclear. Some studies have indicated that ERRα and Hypoxia-inducible factor-1α (HIF1α) are transcription factors that are regulated by phosphorylated ERK and are responsible for initiating the expression of phenotype-related factors (Murray and Huss, 2011; Ning et al., 2014; Masoud and Li, 2015; Kucera et al., 2017; Mylonis et al., 2017). Estrogen-related receptor γ (ERRγ; NR3B3) is a member of the ERR family that also includes the orphan nuclear receptors ERRα (NR3B1) and ERRβ (NR3B2). However, ERRγ is constitutively active due to the active conformation of its ligand-binding domain, even in the absence of a ligand, which differs from other nuclear receptors (Misra et al., 2017).

In this study, we aimed to determine the association between TMJOA and IL-6, ERK, and ERRγ through *in vivo* experiments. Then, *in vitro* experiments were used to verify the regulatory relationship of IL-6 and ERK to ERRγ. U0126 is a phosphorylation inhibitor of ERK and was used to verify that ERRγ is regulated by ERK phosphorylation. GSK5182 is a specific inhibitor of ERRγ that was used to verify whether TMJOA is affected by this reaction after blocking ERRγ activity. Whether ERRγ is a transcription factor that directly binds to the *Mmp9* and *Vegfa* genes was evaluated by chromatin immunoprecipitation (ChIP) assays. In addition, IL-6, U0126, and GSK5182 were added to the chorioallantoic membrane (CAM) model to verify the effects of phospho-ERK (P-ERK) and ERRγ on VEGFA expression and angiogenesis.

## Materials and Methods

### Experimental Induction of TMJOA in Rats

Six-week-old female Wistar rats weighing 190 to 220 g were supplied by the Institute of Shandong University Animal Experimental Center. All animal experiment procedures were performed under guidelines approved by the Institutional Animal Care Committee (protocol GR2018017). The rats were randomly divided into experimental groups (Experimental TMJOA) and control groups (Control). The occlusion disorders were experimentally created by unilateral anterior crossbite (UAC) in the TMJOA groups, as described in previous studies. The rats were anesthetized with 1% pentobarbital sodium (0.30 ml/100 g weight) (Beyotime, China). In the UAC groups, a section of metal tube cut down from a pinhead (length = 2 mm, inner diameter = 2.5 mm) was bonded to the right maxillary incisor, and a curved section of metal tube (length = 4.5 mm, inner diameter = 3.5 mm) was bonded to the right mandibular incisor. A 135° angle leaned to labial side was generated at the end of the tube bonded to the mandibular incisor to create a crossbite relationship between the right incisors. The operation on each rat was completed within 10 min. No loss of the metal tube was observed during the experimental period. Rats in the control groups underwent all procedures described above, but no metal tube was bonded. Experimental TMJOA animals, together with their age-matched controls, were sacrificed at the end of the 8th week, and all animals received the same standardized diet throughout the procedure.

### Isolation and Culture of Rat MCCs

TMJ cartilage tissues were harvested from 4-week-old Wistar rats. Tissues were washed three times with phosphate-buffered saline (PBS), finely minced, digested with 0.25% trypsin for 10 min, and then digested with 0.1% collagenase II (Cell Signaling Technology, USA) in DMEM, supplemented with 20% FBS, 100 mg/ml streptomycin, and 100 mg/ml penicillin. After an incubation at 37°C in a humidified atmosphere containing 5% CO_2_, chondrocytes were collected by centrifugation at 2-h intervals. Next, the cells were resuspended in a 6-cm culture dish with medium. For the duration of the culture, the medium was changed every 3 days. At 48 h after primary cell seeding, the chondrocytes were arranged in a pattern resembling paving stones, and individual chondrocytes exhibited a polygonal shape. The production of collagen II and chondroitin sulfate decreased significantly. As a result, the cells were used at the second passage (P2) in subsequent experiments. Each *in vitro* experiment was repeated three times, and the MCCs used in the three replicate experiments were all from the same rat.

### Cell Proliferation Assay

Condylar chondrocyte proliferation was evaluated using the CCK-8 assay. Second-passage (P2) MCCs were seeded in 96-well plates at a density of 2 × 10^3^ cells/well. Each day, the medium in each well was replaced with 100 μl of fresh medium and 10 μl of CCK-8 reagent (Dojindo, Kumamoto, Japan). After a 3-h incubation at 37°C in a humidified atmosphere containing 5% CO_2_, the results were quantified using a spectrophotometer to measure the absorbance at a wavelength of 450 nm.

### Immunohistochemistry and Safranin O-Fast Green Staining

Rat mandibular condyle cartilage tissues (n = 24, CON: UAC = 12:12) were fixed with 4% paraformaldehyde, decalcified with buffered EDTA (12.5% EDTA, pH 7.4), and embedded in paraffin. The paraffin specimens were cut into 5-μm thick sections. The tissue sections were deparaffinized in xylene solution and rehydrated in a graded series of ethanol solutions. Sections of damaged cartilage from UAC rats and normal cartilage were stained with Safranin O and counterstained with Fast Green. For IHC, after a 3% H_2_O_2_ treatment and antigen retrieval, all tissue sections were blocked at room temperature with 5% bovine serum albumin for 30 min and then incubated with the following primary antibodies at the indicated dilutions: anti-ERRγ antibody (mouse; sc-393969; 1:100; Santa Cruz); anti–phospho-ERK antibody (rabbit; #4370; 1:250; Cell Signaling Technology); anti-MMP9 antibody (rabbit; ab38898; 1:100; Abcam); and anti-VEGFA antibody (rabbit; ab1316; 1:100; Abcam). After sequential incubations with a biotinylated secondary antibody (BOSTER, SA1022, China) and horseradish peroxidase-conjugated avidin (BOSTER, SA1022, China), proteins were labeled with a Diaminobenzidine Substrate kit (BOSTER, SA2022, China).

### Toluidine Blue Staining

MCCs were cultured on 6-well plates for at least 24 h. We analyzed the effects of ERRγ–small interfering RNA (siRNA), ERRγ-cDNA, U0126, and GSK5182 on P2 MCCs that had been treated with recombinant rat IL-6 (20 ng/ml) for 12 h to investigate the mechanisms of ERK and ERRγ in chondrocytes undergoing inflammatory reactions. MCCs were washed with PBS, fixed with paraformaldehyde for 15 min at room temperature, and then stained with 1% toluidine blue (Sigma) for 10 min at 37°C. Next, the stained chondrocytes were washed with distilled water and then imaged. The morphology of MCCs was observed under a microscope.

### Immunofluorescence Staining

MCCs were cultured in 6-well plates for at least 24 h. After the administration of the treatment corresponding to the experimental design, MCCs were washed three times with PBS, fixed with 4% paraformaldehyde for 15 min at room temperature, and then blocked with PBS containing 0.1% Triton X-100 and 1% normal goat serum for 30 min. Fixed chondrocytes were washed with PBS and incubated overnight at 4°C with a polyclonal anti-COL2 (Collagen Type II) antibody (rabbit; 1:100; SAB4500366, Sigma), anti-AGG (Aggrecan) antibody (rabbit; 1:50; ab36861, Abcam) and anti-ERRγ antibody (mouse; sc-393969; 1:100; Santa Cruz). The cells were washed three times, incubated with rhodamine- or fluorescein-conjugated secondary antibodies, and washed again. The chondrocyte nuclei were identified by staining the cells with 4,6-diamidino-2-phenylindole for 1 min.

### Reverse Transcription–Quantitative PCR Analysis

Total RNA was extracted from tissues or chondrocytes using TRIzol reagent (Invitrogen). One microgram of total RNA was reverse transcribed, and reverse transcription–quantitative PCR (RT-qPCR) was performed using a primeScript RT reagent kit (TaKaRa, Japan) according to the manufacturer’s instructions. RT-qPCR was performed using an Applied Biosystems 7500 Real-Time PCR system (ThermoFisher, USA) with the following settings: 10 min of preincubation at 95°C, followed by 40 cycles of 20 s at 95°C and 60 s at 55°C. After each reaction, the cycle threshold (Ct) was recorded when the amplification curve reflected the exponential kinetic measurements. The 2^ΔΔCt^ method was adopted, and glyceraldehyde-3-phosphate dehydrogenase (GADPH) and β-actin served as the reference mRNA and control mRNA, respectively. The primers used to amplify the target genes and β-actin and GADPH are listed in **Table S1**.

### Western Blotting

Cells were collected from 6-well plates and washed with ice-cold PBS. Next, tissue and cells were lysed using radio immunoprecipitation assay lysis buffer containing 1% phenylmethanesulfonyl fluoride. The protein concentrations in the tissue and cell lysates were quantified using a BCA assay kit, and equal amounts of proteins were separated using sodium dodecyl sulfate–polyacrylamide gel electrophoresis and then transferred onto a polyvinylidene fluoride (PVDF) membrane (Millipore, Billerica, MA, USA). The PVDF membrane was blocked with 5% nonfat dry milk in Tris-buffered saline containing 0.1% Tween 20 at room temperature for 30 min and incubated with antibodies against rat antigens. The following antibodies were used to detect the proteins: anti-ERRγ (mouse; sc-393969; 1:500; Santa Cruz); anti-ERRα (rabbit; ab76228; 1:2,500; Abcam); anti-HIF1α (rabbit; ab51068; 1:1,000; Abcam); anti-phospho-ERK (rabbit; #4370; 1:1,000; Cell Signaling Technology); anti-ERK (rabbit; #4695; 1:1,000; Cell Signaling Technology); anti-COL2 polyclonal (rabbit; 1:400; SAB4500366, Sigma); anti-AGG (rabbit; 1:1,000; SAB4500662, Sigma); anti-MMP3 (rabbit; ab52915; 1:5,000; Abcam); anti-MMP13 (rabbit; ab39012; 1:3,000; Abcam); anti-MMP9 (rabbit; ab38898; 1:3,000; Abcam); and anti-VEGFA (rabbit; ab1316; 1:2,000; Abcam). A monoclonal mouse β-actin antibody (1:1,000; Beyotime) was used to detect the level of the β-actin protein as a loading control. Blots were developed by incubating them with a horseradish peroxidase-conjugated secondary antibody (Beyotime) and enhanced chemiluminescence substrate using an Immobilon™ Western Chemiluminescent HRP Substrate kit (Millipore). Blots were exposed to X-ray film for 15 s for detection.

### Plasmids and siRNAs

For the knockdown of ERRγ, ERRα, HIF1α, or ERK expression, siRNAs against ERRγ (ERRγ-siRNA), ERRα (ERRα-siRNA), HIF1α (HIF1α-siRNA), ERK (ERK -siRNA), and the negative control siRNA (CON-siRNA) were prepared (Genechem, Shanghai, China). Rat MCCs were seeded in 12-well plates at a density of 7 × 10^4^ cells/well. After 24 h, a 100-nM siRNA mixture (containing three different target sequence) or si-NC was transiently transfected into the cells with Lipofectamine 2000 (Thermo Fisher, catalog 11668-019, 3 μl/well), and the knockdown efficiency was determined using Western blotting at 48 h after transfection. Rat MCCs were seeded in 6-well plates at a density of 2 × 10^5^ cells/well. After 24 h, the pcDNA3.1-ERRE or ERK vector (2 μg/well) or pcDNA3.1 empty vector (2 μg/well) was transiently transfected into cells using Lipofectamine 2000 (Thermo Fisher, catalog 11668-019, 4 μl/well). The sequences for siRNA are listed in **Table S2**.

### CAM Assay

Fertilized chicken eggs were purchased from Merial-Vital Company (Beijing, China). Eggs shells were cleaned with 75% ethanol and incubated for 8 days at a temperature of 37 ± 0.5°C and a relative humidity of 50% to 80%. Then, the shell was cut and removed to create a small window (1 × 1 cm) above the air chamber. U0126 (50 μM), GSK5182 (50 μM), IL-6 (20 ng/ml), or DMSO was added to the air chamber, and then the eggs were sealed with sterile medical tape and incubated for 48 h. The CAM vasculature was subsequently examined under a microscope (Olympus SZX16). Vessel density was recorded as the percentage of blood vessel area in the whole area compared with the DMSO group using an Image-Pro Plus 6.0 analysis system.

### ChIP Assay

Rat MCCs were seeded in 10-cm^2^ plates at a density of 1 × 10^6^ cells and stimulated with IL-6 (20 ng/ml) for 12 h. The cells were washed three times with ice-cold PBS. DNA and proteins were cross-linked by incubating cells with 1% formaldehyde for 10 min. Excess formaldehyde was quenched by incubating the cells with glycine for 5 min. The cells were lysed, and the nuclei were digested using micrococcal nuclease. Sheared chromatin was diluted and immunoprecipitated with 2 μg of an anti-ERRγ or control IgG antibody. DNA–protein complexes were eluted from Protein A/G agarose beads using a spin column, and the cross-links then were reversed by an incubation with NaCl at 65°C. ChIP assays were performed using a Chromatin Immunoprecipitation kit (Millipore). The relative binding of ERRγ to the ERRE regions of *Mmp9* and *Vegfa* promoters was analyzed using PCR with an Applied Biosystems Simpliamp instrument (ThermoFisher, USA). The primers for ChIP assays were designed to amplify two different ERRE containing regions of the *Mmp9* promoter and regions of the *Vegfa* promoter (**Table S3**). Agarose gel electrophoresis was used to detect DNA–protein binding and molecular weight of the precipitated DNA. For quantitative ChIP assays, RT-qPCR was performed using SYBR premixExTaq reagents (TaKaRa Bio) and an Applied Biosystems 7500 Real-Time PCR system (ThermoFisher, USA).

### Statistical Analysis

All data are presented as the mean ± standard error. Experimental data were analyzed using one-way analysis of variance. Relative indices were analyzed using SPSS version 22.0 software (SPSS, Chicago, IL, USA). The Student-Newman-Keuls q test was used to calculate differences between the groups. The data were graphically presented using GraphPad Prism 7 (San Diego, CA, USA). A P-value of less than 0.05 was considered significant.

## Results

### Relationships Among IL-6, ERK1/2, ERRγ, and TMJOA

**Figure 1:**
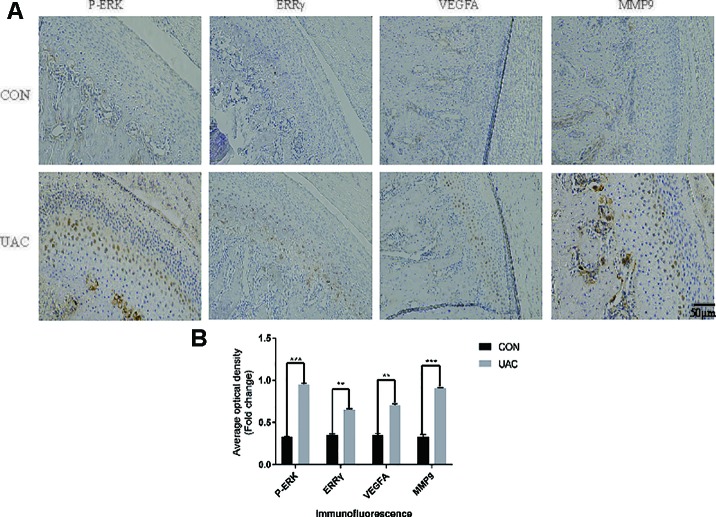
Unilateral anterior crossbite (UAC) induced experimental (TMJOA) in rats, and (ERRγ) expression was upregulated in TMJOA cartilage in rat models. **(A)** Immunostaining for P–ERK, ERRγ, VEGFA and MMP9 was increased in the chondrocyte layer of the UAC group (n = 12 rats per group). **(B)** All experiments were performed in triplicate, and the results are expressed as the mean ± SD. *P < 0.05; **P < 0.01; ***P < 0.005. NS not significant. Scale bar: 50 μm.

**Figure 2:**
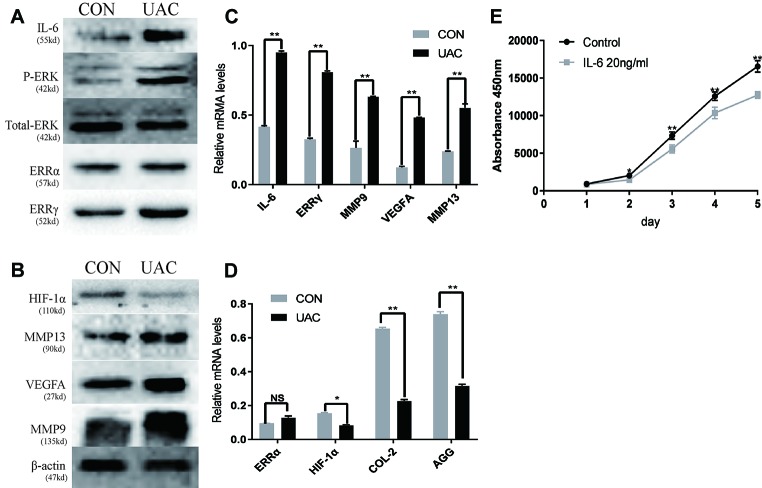
The relationship between interleukin 6 (IL-6), extracellular signal–regulated kinase (ERK), estrogen-related receptor γ (ERRγ) and temporomandibular joint osteoarthritis (TMJOA). **(A)** and **(B)** Representative images of Western blot assays demonstrate the relationship between IL-6, phospho-ERK (P-ERK), ERRγ, matrix metalloproteinase 9 (MMP9), MMP13, HIF-1α, vascular endothelial growth factor A (VEGFA), and TMJOA, while the expression levels of total ERK and β-actin were the same in the cartilage tissues of the control group and the UAC group (n = 12 rats per group). **(C)** and **(D)** RT-qPCR analysis of IL-6, ERRγ, MMP9, MMP13 VEGFA, COL2, AGG, HIF-1α, and ERRα in cartilage tissues of the control group and the UAC group (n = 12 rats per group). **(E)** The CCK-8 assay was used to examine the cell proliferation of each group; the absorbance was measured at 450 nm (n = 3). All experiments were performed in triplicate, and the results are expressed as the mean ± SD. *P < 0.05; **P < 0.01. NS not significant. Scale bar: 50 μm.

We explored the possible association of ERRγ with TMJOA pathogenesis and detected a significant increase in the expression of P-ERK, ERRγ, MMP9, and VEGFA in the rat TMJOA model using immunohistochemical staining (**Figures 1A, B**). We removed the TMJ fibrocartilage and hyaline cartilage from the UAC group and control group and incubated the samples with protein lysis buffer and TRIzol reagent. We detected the expression of IL-6, Phospho-ERK, total ERK, ERRγ, ERRα, HIF1α, MMP13, VEGFA, MMP9, COL2, and AGG using Western blotting to determine the expression patterns of the target proteins, as shown in **Figures 2A, C**. Consistent with the increased protein levels, the RT-qPCR assays revealed marked increases in the mRNA expression levels of IL-6, MMP13, VEGFA, and MMP9 in chondrocytes from the cartilage of rats with TMJOA (**Figures 2B, D**). Densitometric analysis of Western blotting were supplied in **Figure S1**. Because changes in the levels of the ERRα mRNA were not significant, its expression level was negligible compared with that of ERRγ. However, the levels of the COL2 and AGG mRNAs were noticeably reduced in the UAC model group compared with the control group. Similarly, the levels of ERRγ, but not HIF1α or ERRα, were significantly increased in cartilage from an experimental rat model of TMJOA induced by UAC. Interestingly, HIF1α levels were decreased, and MMP13 levels were simultaneously increased, similar to a previous study. Negative feedback modulation between HIF1α and MMP13 levels was observed in OA.

### IL-6 Inhibits Chondrocyte Proliferation and Regulates the Expression of ERRγ, MMP9, and VEGFA

**Figure 3:**
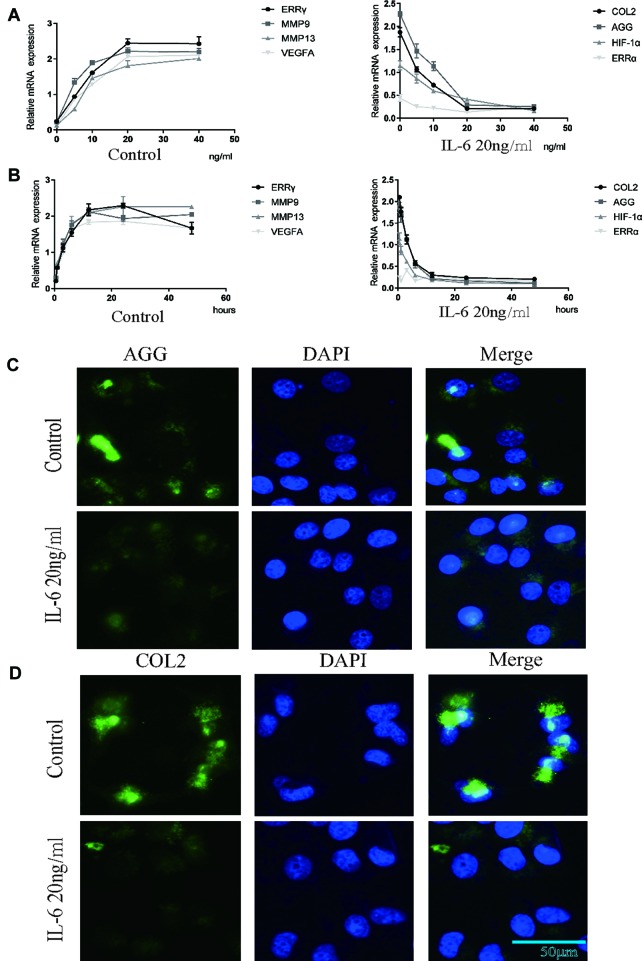
Estrogen-related receptor γ (ERRγ) is the downstream target of the MAPK/extracellular signal–regulated kinase (ERK) pathway that promotes interleukin 6 (IL-6)–induced matrix metalloproteinase 9 (MMP9)/vascular endothelial growth factor A (VEGFA) expression. **(A)** and **(B)** RT-qPCR analysis of ERRγ, MMP9, MMP13 VEGFA, COL2, AGG, HIF-1α, and ERRα in chondrocytes treated with IL-6 (n = 3) to confirm the optimal stimulation concentration and time. **(C)** and **(D)** Images of COL2 and AGG immunofluorescence staining in rat mandibular condylar chondrocytes (MCCs) treated with IL-6 (20 ng/ml) for 12 h (n = 3). All experiments were performed in triplicate, and the results are expressed as the mean ± SD. NS not significant. Scale bar: 50 μm.

We investigated the effects of IL-6 protein on chondrocyte proliferation using CCK-8 assays (**Figure 2E**). The results showed that chondrocyte growth was inhibited by IL-6 (20 ng/ml). Through the concentration gradient and time gradient experiments of IL-6, RT-qPCR confirmed that the optimal concentration and time for establishing TMJOA in an *in vitro* model was 20 ng/ml for 12 h, as shown in **Figures 3A, B**. We stimulated rat MCCs with IL-6 and then performed immunofluorescence staining for COL2 and AGG, as shown in **Figures 3C, D**. We observed a relationship between IL-6 and TMJOA in rat TMJOA models *in vitro* and *in vivo*, and thus, we stimulated MCCs with IL-6 (20 ng/ ml) for 12 h to establish an *in vitro* model of TMJOA.

### ERRγ is the Downstream Target of the MAPK/ERK Pathway That Promotes IL-6–Induced MMP9/VEGFA Expression

**Figure 4:**
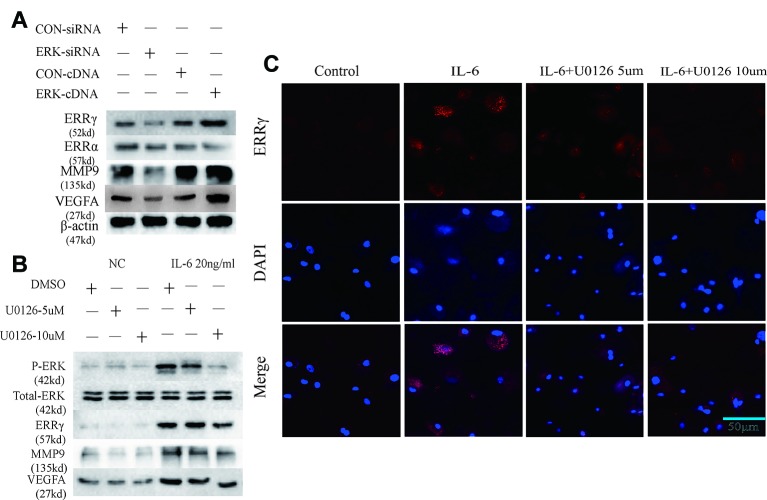
Estrogen-related receptor γ (ERRγ) is a downstream target of the MAPK/extracellular signal–regulated kinase (ERK) pathway and promotes interleukin 6 (IL-6)–induced matrix metalloproteinase 9 (MMP9)/vascular endothelial growth factor A (VEGFA) expression. **(A)** Overexpression and knockdown of ERK altered the levels of the MMP9 and VEGFA proteins induced by ERRγ, but not ERRα (n = 3). **(B)** After IL-6 activated the MAPK/ERK signaling pathway in chondrocytes, changes in the levels of the phospho-ERK (P-ERK), total ERK, ERRγ, MMP9, and VEGFA proteins induced by treatments with different concentrations of the ERK phosphorylation inhibitor U0126 were analyzed (n = 3). **(C)** IL-6 and different concentrations of U0126 regulated the expression of ERRγ, as determined using immunofluorescence staining (n = 3). All experiments were performed in triplicate, and the results are expressed as the mean ± SD. *P < 0.05; **P < 0.01. NS not significant. Scale bar: 50 μm.

ERK was overexpressed in rat MCCs by transfecting a plasmid containing ERK cDNA, and an ERK-specific siRNA was used to knockdown ERK expression. ERK overexpression and knockdown did not alter the expression of ERRα, but ERRγ expression was regulated by changes in total ERK levels, as shown in **Figure 4A**. Similarly, an increasing trend in MMP9 and VEFGA expression was observed in cells transfected with ERK cDNA, and a decreasing trend was observed in ERK-knockdown cells. U0126, a specific inhibitor of the phosphorylation of components of the MAPK/ERK signaling pathway, was used to verify the relationship between phosphorylated ERK and ERRγ. Two concentrations of U0126, 5 and 10 μM, were applied to rat MCCs along with IL-6 to observe changes in the expression of ERRγ/MMP9/VEFGA in cells with identical total ERK levels (**Figure 4B**). Densitometric analysis of Western blotting were supplied in **Figure S2**. ERK phosphorylation was significantly inhibited by U0126, and ERRγ expression decreased significantly. In keeping with this observation, the results from immunofluorescence staining confirmed this conclusion (**Figure 4C**). At the same time, the levels of MMP9 and VEFGA showed a decreasing trend. Based on these results, we suggest that the IL-6/ERK axis contributes to the regulation of ERRγ expression in articular chondrocytes. These results showed that IL-6/ERK/ERRγ play an essential role in OA cartilage destruction by upregulating MMP9 and VEFGA expression.

### ERRγ Plays an Essential Role in Cartilage Destruction in TMJOA by Influencing MMP9/VEGFA/COL2/AGG

**Figure 5:**
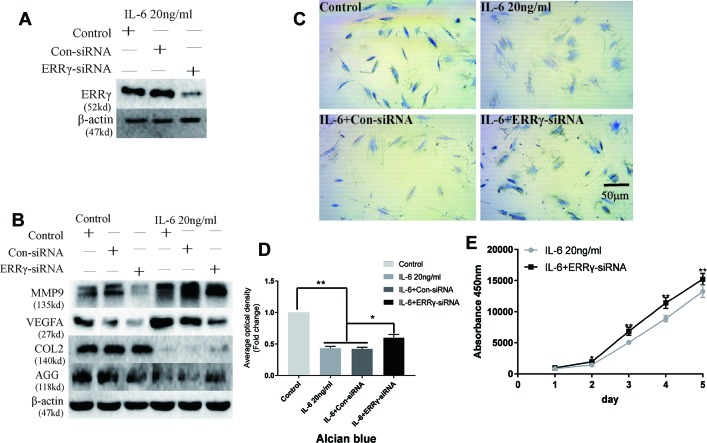
Interleukin 6 (IL-6)–treated chondrocytes increases matrix degradation regulated by estrogen-related receptor γ (ERRγ). **(A)** Non-transfected cells (control group) or cells transiently transfected with the control small interfering RNA (siRNA) (Con-siRNA) or ERRγ-siRNAs were cultured in the presence of IL-6. The Western blot assays showed decreased levels of ERRγ in mandibular condylar chondrocytes (MCCs) transfected with ERRγ-specific siRNAs (n = 3). **(B)** Western blotting show that IL-6-induced increases in matrix metalloproteinase 9 (MMP9) and vascular endothelial growth factor A (VEGFA) were abrogated in ERRγ-knockdown cells and IL-6-induced decreases in COL2 and AGG were reversed by ERRγ-siRNAs (n = 3). **(C)** and **(D)** Toluidine blue staining results of MCCs transfected with 20 nM Con-siRNA or 20 nM ERRγ-siRNA and stimulated by 20 ng/ml IL-6 for 12 h (n = 3). **(E)** ERRγ knockdown enhanced the proliferation of MCCs in medium containing 20 ng/ml IL-6, as determined by CCK-8 assays (n = 3). All experiments were performed in triplicate, and the results are expressed as the mean ± SD. *P < 0.05; **P < 0.01. Scale bar: 50 μm.

The ERR siRNA inhibited ERRγ expression when chondrocytes were stimulated with IL-6, as shown in **Figure 5A**. Similarly, the IL-6-induced upregulation of MMP9 and VEGFA was abrogated by the knockdown of ERRγ with siRNAs, as shown in **Figure 5B**. Densitometric analysis of Western blotting were supplied in **Figure S3**. Meanwhile, the expression of COL2 and AGG decreased in IL-6-stimulated rat MCCs, and the changes were reversed in cells transfected with ERRγ siRNAs. Images and the analysis of toluidine blue staining (presented in **Figures 5C, D**) showed that IL-6 significantly inhibited chondrocyte proliferation. The transfection of the ERRγ siRNA blocked the effects of IL-6 on chondrocyte proliferation (**Figure 5E**).

**Figure 6:**
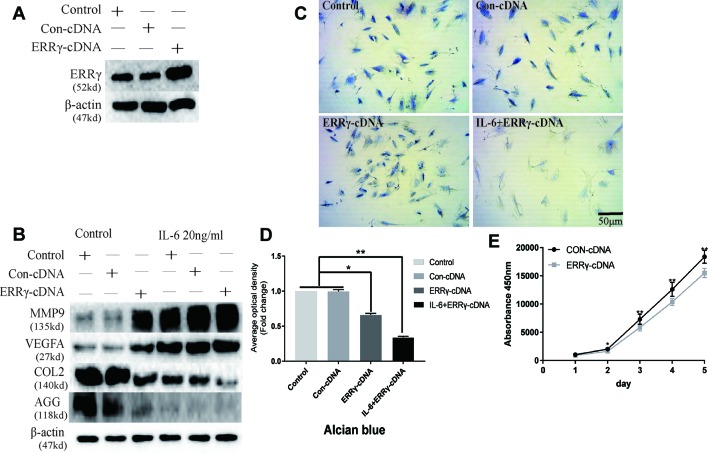
Estrogen-related receptor γ (ERRγ) causes temporomandibular joint osteoarthritis (TMJOA) chondrocyte extracellular matrix degradation and vascularization by directly or indirectly regulating the expression of matrix metalloproteinase 9 (MMP9)/vascular endothelial growth factor A (VEGFA)/COL2/AGG. **(A)** Non-transfected cells (control group) or cells transiently transfected with the pcDNA vector or ERRγ cDNA constructs were cultured in the absence of interleukin 6 (IL-6). Western blots of TMJ cell lysates showed increased levels of ERRγ in cells transfected with the ERRγ cDNA (n = 3). **(B)** Representative images showing that ERRγ overexpression increases the expression of MMP9 and VEGFA in cells treated with or without IL-6 and inhibited the expression of COL2 and AGG (n = 3). **(C)** and **(D)** Toluidine blue staining results of mandibular condylar chondrocytes (MCCs) transfected with 50 nM Con-cDNA or 50 nM ERRγ-cDNA in normal medium or stimulated by 20 ng/ml IL-6 for 12 h (n = 3). **(E)** ERRγ overexpression inhibited the proliferation of MCCs in normal medium, as determined by CCK-8 assays (n = 3). All experiments were performed in triplicate, and the results are expressed as the mean ± SD. *P < 0.05; **P < 0.01. Scale bar: 50 μm.

The transfection of the ERRγ cDNA increased basal ERRγ levels, as shown in **Figure 6A**. We investigated whether the overexpression of ERRγ alone modulated VEGFA, MMP9, COL2, and AGG expression. The overexpression of ERRγ amplified IL-6-mediated increases in VEGFA and MMP9 levels (**Figure 6B**). Densitometric analysis of Western blotting revealed that ERRγ increased the production of matrix-degrading enzymes and angiogenesis factors and reduced the expression of COL2 and AGG in chondrocytes (**Figure S3**). Toluidine blue staining showed varying degrees of dark cytoplasmic staining after ERRγ overexpression, but still maintained their morphology without excessive fibrosis, as shown in **Figures 6C, D**. This observation differs from the stimulatory effect of IL-6 on chondrocytes, indicating that ERRγ may not affect the dedifferentiation of chondrocytes. The transfection of the ERRγ cDNA inhibited the proliferation on chondrocyte (**Figure 6E**).

### ERRγ Directly Activates the Transcription of *Mmp9* and *Vegfa*, and U0126 and GSK5182 Exert Significant Inhibitory Effects on Angiogenesis

**Figure 7:**
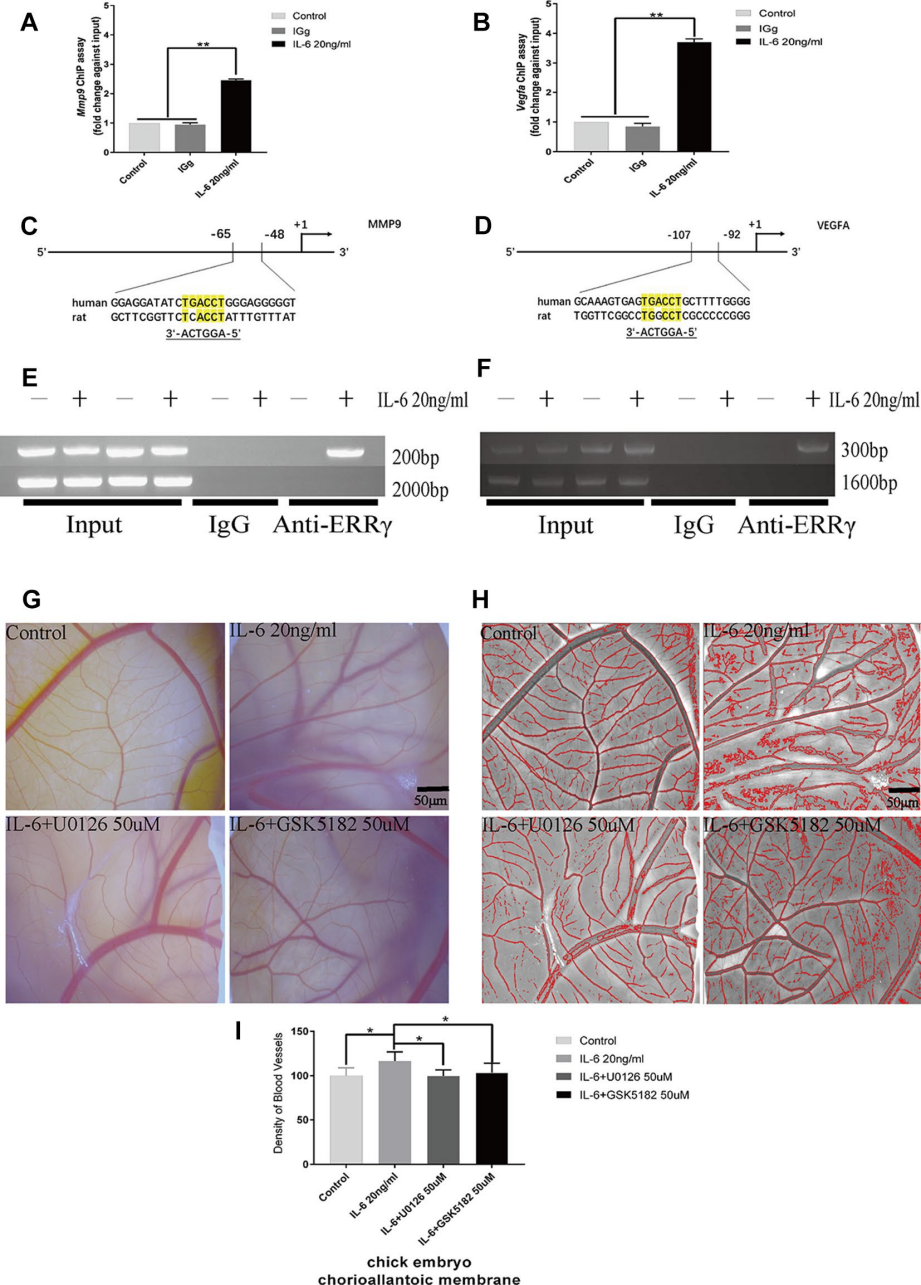
Estrogen-related receptor γ (ERRγ) directly activates the transcription of Mmp9 and Vegfa in mandibular condylar chondrocytes (MCCs), and U0126 and GSK5182 exert significant inhibitory effects on angiogenesis. **(A)** and **(B)** Quantitative Mmp9 and Vegfa chromatin immunoprecipitation (ChIP) assays were performed, and the data are presented as fold changes relative to each input (n = 3). **(C)** and **(D)** The ERRE sequences in the promoter regions of Mmp9 and Vegfa are indicated. **(E)** and **(F)** ChIP assays showing the binding of ERRγ to the Mmp9 and Vegfa promoters. Numbers on the right indicate the distance from the transcription start site. MCCs were treated with interleukin 6 (IL-6) (20 ng/ml) for 12 h, and soluble chromatin was immunoprecipitated with an ERRγ-specific antibody (n = 3). **(G**–**I)** Representative images and pooled data in the graph show the appearance of blood vessels in chick embryo chorioallantoic membranes (CAMs) treated with DMSO, IL-6 (20 ng/ml), IL-6+U0126 (50 μM), or IL-6+GSK5182 (50 μM) for 24 h (n = 3). All experiments were performed in triplicate, and the results are expressed as the mean ± SD. *P < 0.05; **P < 0.01. Scale bar: 50 μm.

Because ERRγ is a member of the orphan nuclear receptor family, its structure and function determine its role in activating the transcription of downstream genes. We identified a conserved ERRγ-responsive element (**Figures 7C, D**) in the human and rat *Mmp9* and *Vegfa* promoters. Rat MCCs in the experimental group were treated with 20 ng/ml IL-6 and fixed with 37% formaldehyde after 6 h. As shown in the results of the ChIP assay presented in **Figures 7A, B**, ERRγ bound the ERRE (AGGTCA) in the rat Mmp9 and Vegfa promoters, indicating that this site likely mediates ERRγ-induced MMP9 and VEGFA transcription (**Figures 7E, F**).

The effects of IL-6, ERRγ, and ERK1/2 on angiogenesis in TMJ cartilage were verified by treating the chick embryo CAM model with U0126 and GSK5182. A significant increase in angiogenesis was observed in the DMSO+IL6 group (P < 0.01), while angiogenesis was inhibited by the addition of the specific inhibitors GSK5182 or U0126 with IL-6 to the CAM (P < 0.01) (**Figures 7G–I**). The above results suggest that ERRγ inhibition not only reduced cartilage destruction in TMJOA models but also inhibited intra-articular angiogenesis. The angiogenesis mediated by ERRγ activation may be widely increased throughout the synovium, osteophytes, and menisci and lead to the ossification of osteophytes and the deep layers of articular cartilage (Mapp and Walsh, 2012). We suggest that the inhibition of ERRγ can protect the TMJ from two functional perspectives, ECM degradation, and angiogenesis.

### Therapeutic Effects of ERRγ siRNAs and Inverse Agonist GSK5182 on TMJOA

**Figure 8:**
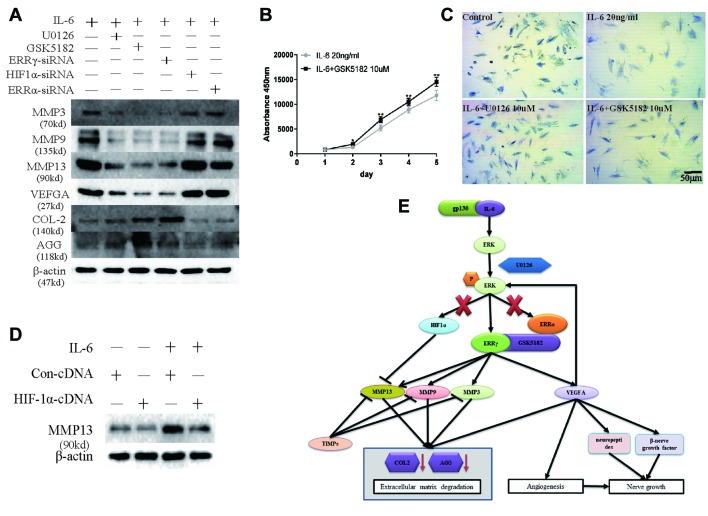
Sufficiency experimental designed to verify the inhibitory effects of Estrogen-related receptor γ (ERRγ) small interfering RNAs (siRNAs), U0126, and GSK5182 on temporomandibular joint osteoarthritis (TMJOA). **(A)** Western blots reveal certain therapeutic effects of U0126, GSK5182, and ERRγ siRNAs on TMJOA, and HIF-1α and ERRα do not reverse the effects of extracellular matrix degradation and vascularization on TMJOA (n = 3). **(B)** CCK-8 assay was used to examine the effect of GSK5182 on cell proliferation (n = 3). **(C)** Images of toluidine blue staining of mandibular condylar chondrocytes (MCCs) (P2) treated with interleukin 6 (IL-6) (20 ng/ml) for 12 h and stimulated with U0126 or GSK5182 (10 μM) for 12 h (n = 3). **(D)** MCCs transiently transfected with pcDNA vector or HIF-1α cDNA constructs were cultured in the absence or presence of IL-6. Western blots of MCC lysates showed a decrease in IL-6-induced matrix metalloproteinase 13 (MMP13) levels in cells overexpressing HIF-1α (n = 3). **(E)** Schematic showing the catabolic effects of the IL6-MAPK/extracellular signal–regulated kinase (ERK)–ERRγ axis on human chondrocytes. All experiments were performed in triplicate, and the results are expressed as the mean ± SD. *P < 0.05; **P < 0.01. NS not significant. Scale bar: 50 μm.

Next, we examined whether ERRγ represents a potential therapeutic target for OA. Because ERK, an upstream signaling component involved in regulating ERRγ expression, upregulates MMP9 and VEGFA expression in chondrocytes, we examined whether the IL-6-induced upregulation of MMP3/9/13 and VEGFA requires the transcriptional activity of ERRγ, ERRα, or HIF1α. We treated IL-6-stimulated chondrocytes with ERRγ, ERRα, or HIF1α siRNAs to validate ERRγ as a potential therapeutic target for OA. Only the ERRγ siRNAs significantly reduced all IL6-induced manifestations of OA, including the upregulation of MMP3/9/13 and VEGFA and the downregulation of COL-2 and AGG (**Figure 8A**). Densitometric analysis of Western blotting were supplied in **Figure S4**. Treatment with GSK5182 significantly inhibited the effects of IL-6 on chondrocyte proliferation (**Figure 8B**). The matrix content of chondrocytes was determined by toluidine blue staining at 12 h after IL-6 stimulation. After image analysis, the mean optical density of the staining under IL-6 stimulation was significantly lower than that of the NC group, and matrix staining was enhanced by U0126 and GSK5182 (**Figure 8C**), suggesting that U0126 and GSK5182 participates in the catabolism pathway induced by IL-6.

However, chondrocytes transfected with HIF1α siRNAs further upregulated the expression of MMP13. Therefore, we transfected the HIF1α cDNA into chondrocytes to verify the effect of HIF1α on MMP13 expression (**Figure 8D**). Densitometric analysis of Western blotting were supplied in **Figure S4**. HIF1α inhibited MMP13 expression in rat MCCs. The overall mechanism identified in our study is illustrated in **Figure 8E**. First, following the binding of IL6 to its receptor gp130, the MAPK/ERK signaling pathway was activated, and transcription factors related to the transcriptional activation of the *Mmp9* and *Vegfa* genes were selected from among three possible downstream transcription factors, ERRα, ERRγ, and HIF1α.

## Discussion

In addition to cartilage degradation and subchondral bone remodeling, cartilage vascularization is an important hallmark of OA. We confirmed high levels of IL-6 expression in the TMJ of a rat TMJOA model (UAC) and revealed that IL-6 activated MCCs to produce MMPs, thereby degrading the cartilage ECM. Histological evidence from patients with OA suggests that synovial inflammation is an important pathological and clinical characteristic required for the diagnosis of OA (Haywood et al., 2003; Benito et al., 2005). Histological evidence of inflammation is characterized by hyperplasia in the synovium, local infiltration of macrophages, and angiogenesis (Smith et al., 1997; Mapp and Walsh, 2012), and the degree of angiogenesis in OA increases with increasing macrophage infiltration and the histological grade of inflammation (Haywood et al., 2003). This increase is likely due to the production of proangiogenic factors such as VEGFA by articular chondrocytes. VEGFA increases the expression of MMPs in TMJ articular chondrocytes by activating the MAPK/ERK pathway, and VEGFA may be involved in osteophyte formation. After osteophyte formation, the sustained production of VEGFA will also promote vascularization within the osteophytes, a process that is often followed by vascularization within the articular cartilage (Withrow et al., 2016). The development of inflammation and angiogenesis suggests that VEGFA is a pro-OA factor that persists in the OA process, including the early, intermediate, and late stages. Inflammation in a bone or joint usually leads to increased osteoclast activity, inducing channels to extend from the subchondral bone into the articular cartilage and eventually pass across the tidemark into normally avascular, noncalcified cartilage (Suri et al., 2007). Blood vessels grow from the subchondral bone to occupy the osteochondral channels and are accompanied by extensions of sympathetic and sensory nerves (Brown and Weiss, 1988; Mapp and Walsh, 2012). Therefore, neovascularization might contribute to the pain experienced by patients with OA because of the accompanying sensory innervation.

IL-6 increased angiogenesis in the CAM model, and we further analyzed the main mechanism by which IL-6 induced angiogenesis. First, IL-6 stimulates chondrocytes to produce proangiogenic factors such as VEGFA. However, IL-6 is a cytokine whose receptor is located on the membrane, indicating that increased VEGFA production is a process mediated by signals transduced through various receptors. The MAPK/ERK signaling pathway is activated by a wide range of inflammatory factors and proangiogenic factors in TMJOA, such as IL-1β, IL-6, and VEGF (Mihara et al., 2012; Feng et al., 2017; Yang et al., 2017; Nonaka et al., 2018; Wu et al., 2018). Thus, ERK phosphorylation may play a vital role in the production and effectiveness of angiogenic factors. The present study provided evidence to support a novel critical homeostatic mechanism by which ERRγ functions as a key regulator of cartilage degradation and angiogenesis through an ERK-mediated signaling pathway in mandibular condylar chondrocytes.

Chondrocyte extracellular matrix degradation and angiogenesis contribute to osteochondral damage and disc pathology in patients with OA. A balance between matrix-degrading enzymes and factors that inhibit these enzymes and between pro-angiogenic factors and anti-angiogenic factors exists in normal joints, and excessive production of inflammatory factors in patients with OA disrupts this balance (Bonnet and Walsh, 2005). The suppression of ERRγ expression or transcriptional activity decreases the levels of the MMP3/9/13 and VEGFA proteins to subsequently inhibit extracellular matrix degradation and vascularization and reduce the extent of cartilage destruction. Inhibition of ERRγ expression in MCCs transfected with ERRγ siRNAs decreased MMPs and VEGFA expression. Based on the experimental evidence, ERRγ is a transcription factor that directly regulates the expression of the *Mmp9* and *Vegfa* mRNAs. Two inhibitors, U0126 and GSK5182, restrained the production of downstream MMPs and VEGFA and significantly decreased angiogenesis in the CAM model. ERRγ may be a key factor that simultaneously inhibits metalloproteinase and vascular endothelial growth factor production following IL-6-induced inflammation. Based on the results from the present study, the inhibition of ERRγ expression by ERRγ siRNAs or inhibition of ERRγ transcriptional activity by GSK5182 ultimately inhibited the expression of MMP9 and VEFGA, and we provided direct evidence that ERRγ induces *Mmp9*/*Vegfa* gene expression.

In addition to ERRγ, other members of the ERR family are associated with TMJOA, as ERRα mRNA and protein were detected in rat MCCs. 17-β Estradiol (E2) controls MCC proliferation *via* ERRα, and the overexpression of ERRα increased the levels of the Sox9 and GDF−5 mRNAs and proteins (Ahmad et al., 2018). Ultimately, ERRα exerted an important regulatory effect on the proliferation and differentiation of MCCs from female rats *in vitro*. However, in the rat UAC model, namely, the *in vivo* rat TMJOA model, no significant difference in ERRα expression was observed compared with the control group. The MAPK/ERK pathway does not regulate ERRα expression in the *in vitro* TMJOA model. We hypothesized that IL-6 does not regulate the expression of ERRα through the MAPK/ERK pathway and that ERRα expression may be inhibited in an inflammatory environment.

## Data Availability Statement

The datasets analyzed in this manuscript are not publicly available. Requests to access the datasets should be directed to 168277500@qq.com.

## Ethics Statement

The animal study was reviewed and approved by Institutional Animal Care Committee (protocol GR2018017).

## Author Contributions

Conceived and designed the experiments: HaZ, SL, CM. Performed the experiments: HaZ, SL, SM, LY. Analyzed the data: HaZ, GC. Contributed reagents, materials, and analysis tools: LC, CM, HuZ, ZM.

## Funding

This work was supported by National Natural Science Foundation of China (61771290, 61871393), the Science and Technology Development Plans of Shandong province (Grant 2018GSF118196), Taishan Scholars (tsqn201812137), Natural Science Foundation of Shandong Province (No. ZR2018PH022, ZR2019PH015), and China Postdoctoral Science Foundation (No. 2019M652408).

## Conflict of Interest

The authors declare that the research was conducted in the absence of any commercial or financial relationships that could be construed as a potential conflict of interest.

## Supplementary Material

Supplementary Table S1 Primer sequences for RT-qPCR.

Supplementary Table S2 Sequences for siRNA.

Supplementary Table S3 Primer sequences and conditions for ChIP assay.

Supplementary Figure S1 Densitometric analysis of western blots in **Figure 2a and b** and Supplementary figure for **Figure 2a**.

Supplementary Figure S2 Densitometric analysis of western blots in **Figure 4a** and Densitometric analysis of western blots in **Figure 4b**.

Supplementary Figure S3 Densitometric analysis of western blots in **Figure 5b** and Densitometric analysis of western blots in **Figure 6b**.

Supplementary Figure S4 Densitometric analysis of western blots in **Figure 8a** and Densitometric analysis of western blots in **Figure 8d**.
