# Supplementary material for: Estrogen-Related Receptor γ Induces Angiogenesis and Extracellular Matrix Degradation of Temporomandibular Joint Osteoarthritis in Rats
Source: Front Pharmacol. 2019 Nov 6;10:1290. doi: 10.3389/fphar.2019.01290 (PMC6851845; doi:10.3389/fphar.2019.01290)
Supplement: Supplementary Table S1 — Primer sequences for RT-qPCR. [file Table_1.pdf]

# Supplementary Table S1: Primer sequences for RT-qPCR

| Gene | Forward(5'-3') | Reverse(5'-3') |
|---|---|---|
| ERRγ | GCGTAGAATAGATGCTGAG | CCAATGATAACCACCAACT |
| ERRα | CATCTGCTGGTGGTTGAA | AGAGTGACAGTGAGGAGAA |
| HIF1α | CTCCATTACCTGCCTCTG | ATTCTTCGCTTCTGTGTCT |
| MMP13 | ATGTGGAGTGCCTGATGTG | AAGCGTGTGCCAGAAGAC |
| VEGFA | CCGGTTTAAATCCTGGAGCG | TTTAACTCAAGCTGCCTCGC |
| MMP9 | ACTGCTGGTCCTTCTGAG | ATTGGCTTCCTCCGTGAT |
| COL2 | AAGAGCAAGGAGAAGAAG | TTACAGTGGTAGGTGATG |
| AGG | GCAGCACAGACACTTCAGGA | CCCACTTTCTACAGGCAAGC |
| β-actin | CGGTCAGGTCATCACTAT | TGTTGGCATAGAGGTCTT |
| GADPH | ATGATTCTACCCACGGCAAG | CTGGAAGATGGTGATGGGTT |
