# Supplementary material for: Estrogen-Related Receptor γ Induces Angiogenesis and Extracellular Matrix Degradation of Temporomandibular Joint Osteoarthritis in Rats
Source: Front Pharmacol. 2019 Nov 6;10:1290. doi: 10.3389/fphar.2019.01290 (PMC6851845; doi:10.3389/fphar.2019.01290)
Supplement: Supplementary Table S2 — Sequences for siRNA. [file Table_2.pdf]

Supplementary Table S2: Sequences for siRNA

Gene	5'-3'
ERK-siRNA	CCTCAAAGCAATAGCTCTT
ERRγ-siRNA	GAAAGAGCTTCTGCACAAT
ERRα-siRNA	AGCGCAAGGGCCTCAATTA
HIF1α-siRNA	GCAAGGCCTTACATGTTAA
