# Supplementary material for: Estrogen-Related Receptor γ Induces Angiogenesis and Extracellular Matrix Degradation of Temporomandibular Joint Osteoarthritis in Rats
Source: Front Pharmacol. 2019 Nov 6;10:1290. doi: 10.3389/fphar.2019.01290 (PMC6851845; doi:10.3389/fphar.2019.01290)
Supplement: Supplementary Table S3 — Primer sequences and conditions for ChIP assay. [file Table_3.pdf]

Supplementary Table S3: Primer sequences and conditions for ChIP assay

Target	Strand	Primer sequences	Size (bp)	AT (°C)	Origin
MMP9#1	Forward	5'CACCATGCGTACCCTCCTTCC 3'	135	60	Rat
	Reverse	5' ATGCCCTGCCCACAGTCCC 3'			
MMP9#2	Forward	5' GTGGACCCAGGACTGCAAT 3'	67	60	Rat
	Reverse	5' GCTCCCCGGCTTCTCTTATAT 3'			
MMP9#3	Forward	5' CGATGTTAGCCAGCCTGAGAA 3'	132	60	Rat
	Reverse	5' GCTCTTTGAAGCAGGATTTGG 3'			
VEGFA#1	Forward	5' AGCCTTACCTCTACTCCCATCC 3'	91	60	Rat
	Reverse	5' TTCAAACCTACCCTAGCATTCA 3'			
VEGFA#2	Forward	5' CCTCCGGGCCACTGACTAA 3'	74	60	Rat
	Reverse	5'CTCTGGAGAGGATATGGCATCA 3'			
VEGFA#3	Forward	5'GATGGAGATGGTGGAGTAACCT 3'	129	60	Rat
	Reverse	5' GCTCTGATACCTGTGGGAAGA 3'			
