# Supplementary figures and images for: Estrogen-Related Receptor γ Induces Angiogenesis and Extracellular Matrix Degradation of Temporomandibular Joint Osteoarthritis in Rats
Source: Front Pharmacol. 2019 Nov 6;10:1290. doi: 10.3389/fphar.2019.01290 (PMC6851845; doi:10.3389/fphar.2019.01290)

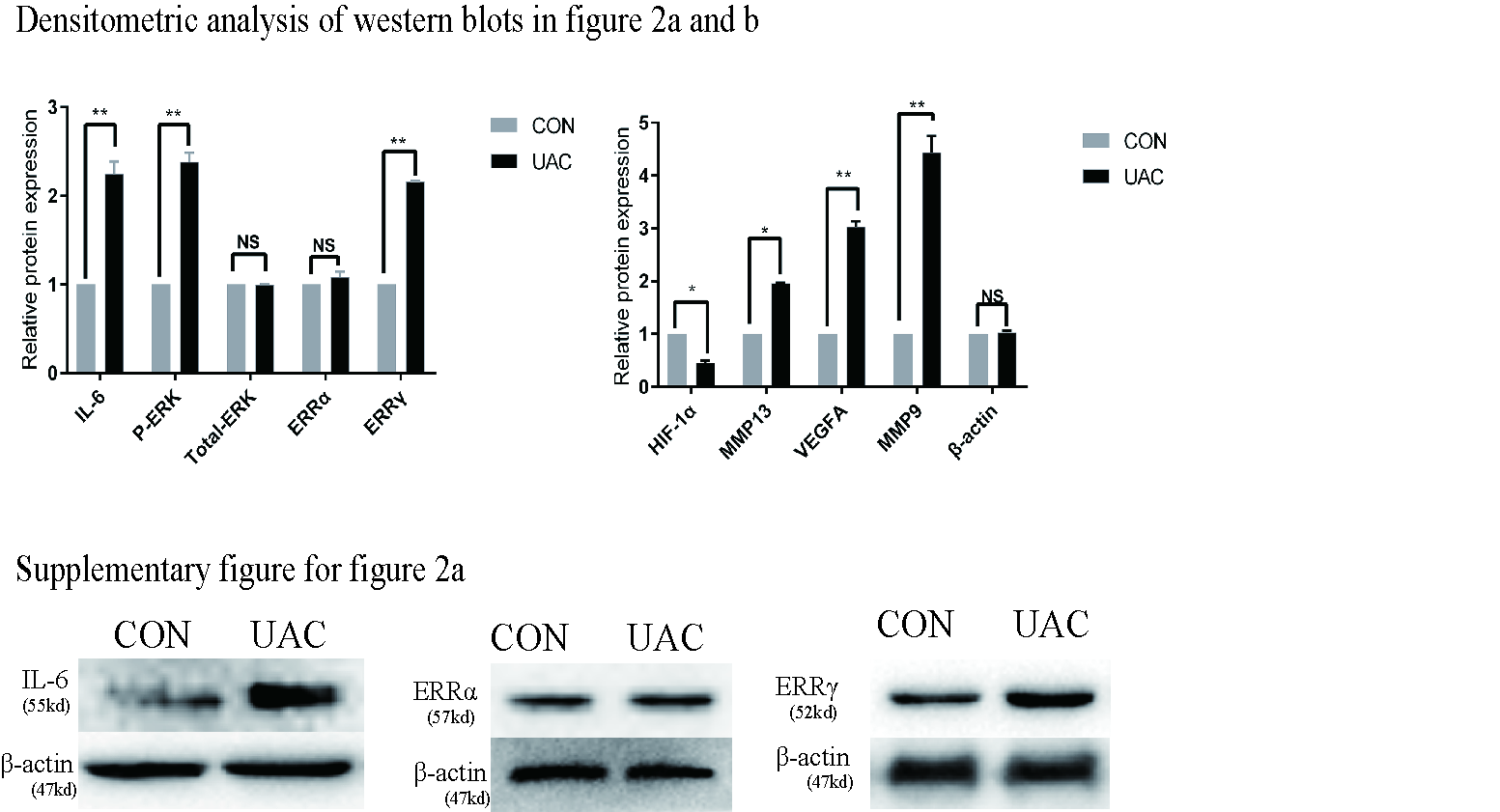

Supplement: Supplementary Figure S1 — Densitometric analysis of western blots in Figure 2a and b and Supplementary figure for Figure 2a . [file Image_1.tif]

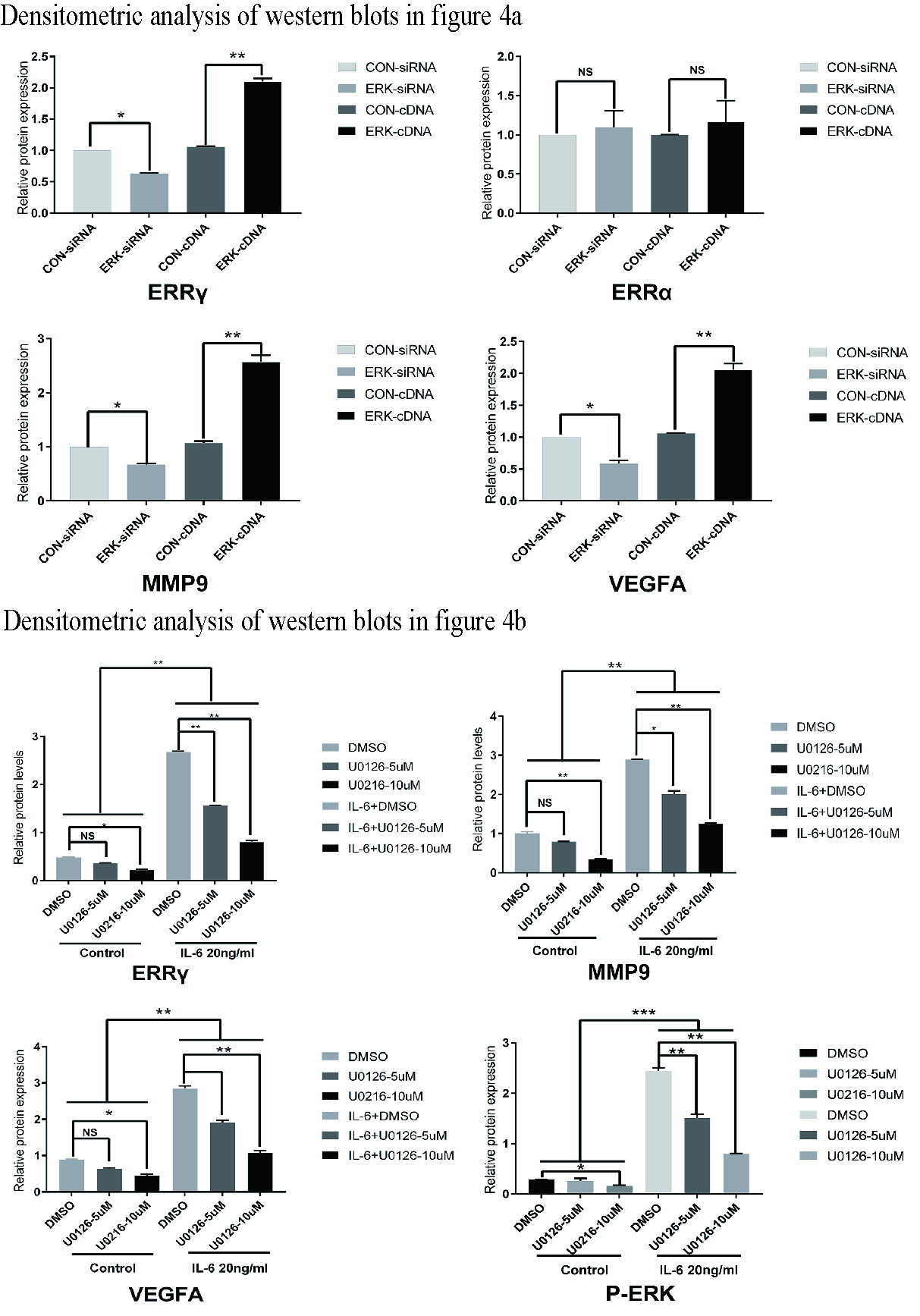

Supplement: Supplementary Figure S2 — Densitometric analysis of western blots in Figure 4a and Densitometric analysis of western blots in Figure 4b . [file Image_2.tif]

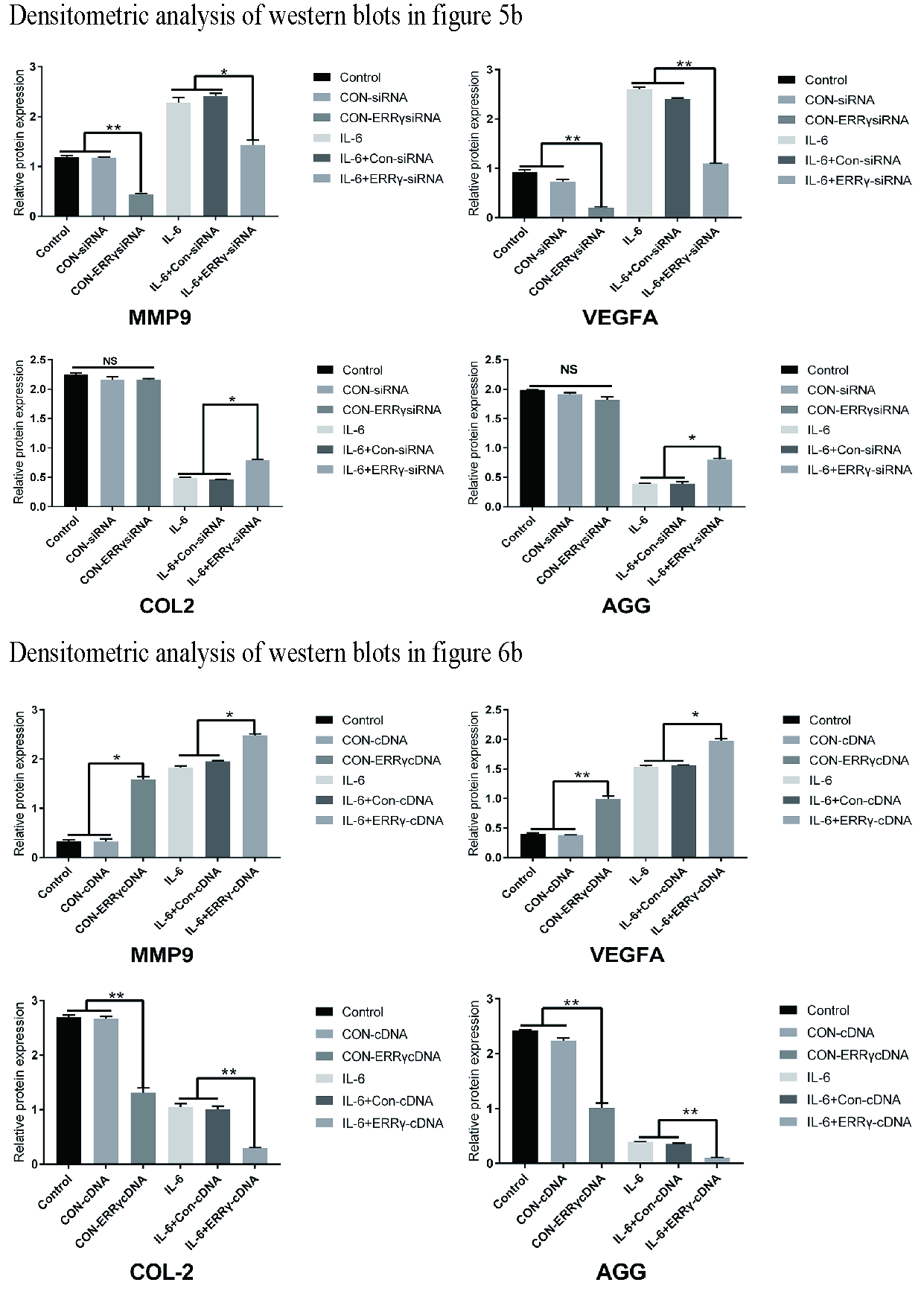

Supplement: Supplementary Figure S3 — Densitometric analysis of western blots in Figure 5b and Densitometric analysis of western blots in Figure 6b . [file Image_3.tif]

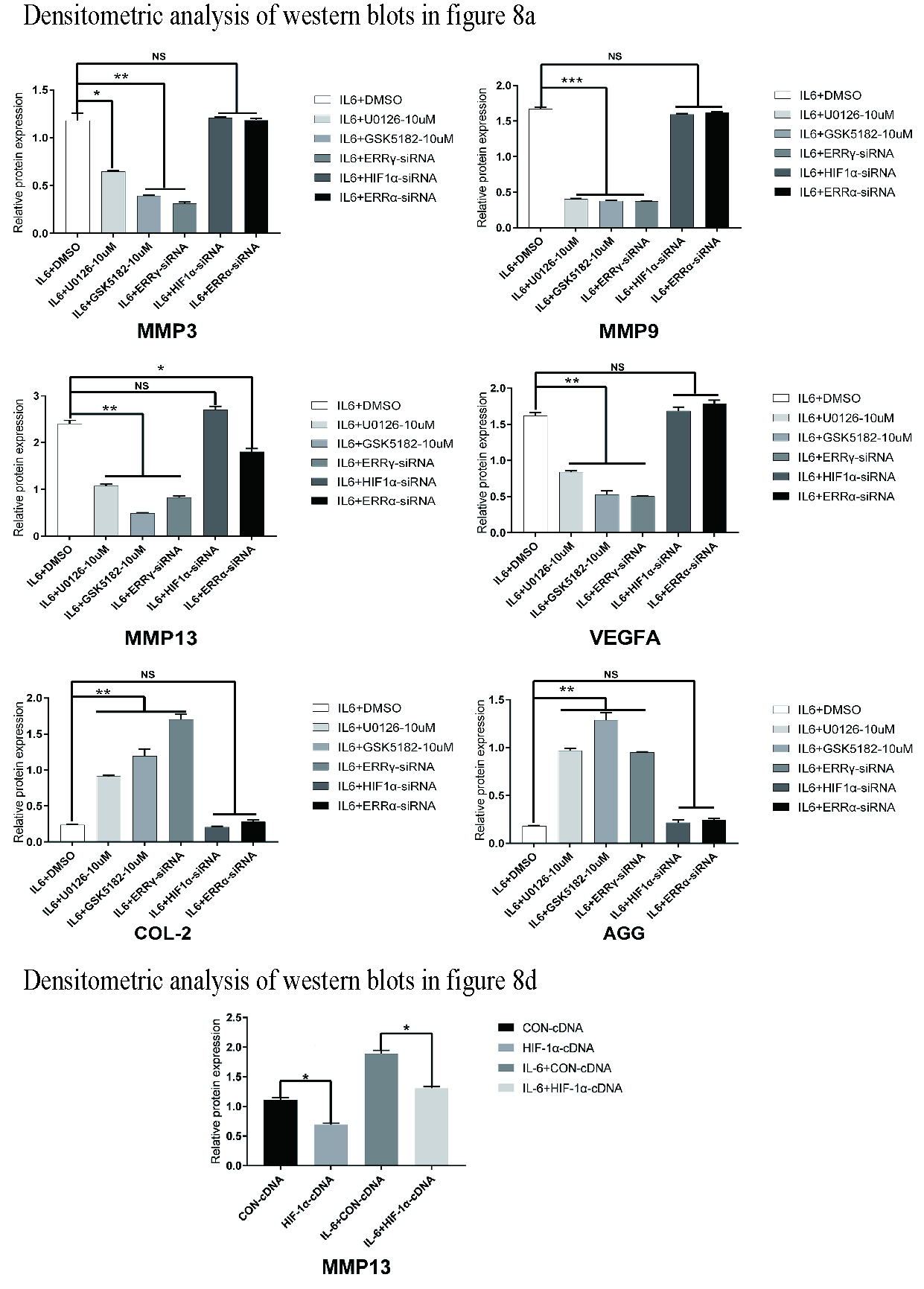

Supplement: Supplementary Figure S4 — Densitometric analysis of western blots in Figure 8a and Densitometric analysis of western blots in Figure 8d . [file Image_4.tif]
